# Regulation of Neural Circuitry under General Anesthesia: New Methods and Findings

**DOI:** 10.3390/biom12070898

**Published:** 2022-06-28

**Authors:** Kai Zhang, Jiacheng Pan, Yonghao Yu

**Affiliations:** 1 Department of Anesthesiology, Tianjin Medical University General Hospital, Tianjin 300052, China; zhangkai5z@126.com (K.Z.); panjiacheng0608@126.com (J.P.); 2 Tianjin Institute of Anesthesiology, Tianjin 300052, China

**Keywords:** general anesthesia, neural nuclei and circuits, in vivo calcium imaging, chemogenetics, optogenetics

## Abstract

General anesthesia has been widely utilized since the 1840s, but its underlying neural circuits remain to be completely understood. Since both general anesthesia and sleep are reversible losses of consciousness, studies on the neural-circuit mechanisms affected by general anesthesia have mainly focused on the neural nuclei or the pathways known to regulate sleep. Three advanced technologies commonly used in neuroscience, in vivo calcium imaging, chemogenetics, and optogenetics, are used to record and modulate the activity of specific neurons or neural circuits in the brain areas of interest. Recently, they have successfully been used to study the neural nuclei and pathways of general anesthesia. This article reviews these three techniques and their applications in the brain nuclei or pathways affected by general anesthesia, to serve as a reference for further and more accurate exploration of other neural circuits under general anesthesia and to contribute to other research fields in the future.

## 1. Introduction

General anesthesia has been used since the 1840s and is a necessary safety guarantee for most surgeries today. From destroying the lipid bilayer on the cell surface [1] to protein targets [2] and the specific molecular sites on specific receptors [3], the mechanisms of general anesthetics have been well documented. However, the neural circuits underlying general anesthesia remain relatively unclear, compared with the protein and molecular targets of general anesthetics. Growing evidence suggests that many neural circuits that regulate the sleep–wake cycle are involved in the general-anesthesia effect as well. For example, varieties of neurons and their projections that are important to promote wakefulness, including monoaminergic [4,5], cholinergic [6], glutamatergic [7], and orexinergic neurons [8], participate in promoting emergence from general anesthesia. The γ-aminobutyric acid (GABA) neurons in the ventrolateral preoptic nucleus (VLPO) extensively innervate and suppress multiple arousal-promoting brain regions [9,10]. The VLPO is vital for both the initiation and maintenance of sleep [11]. It is reported that VLPO is necessary for propofol-induced inhibition of locus coeruleus (LC) activity [12]. Whereas directly specific activation of GABAergic in the VLPO modulates sleep–wake architecture but not anesthetic-state transitions [13]. Therefore, although there may be some common pathways between general anesthesia and sleep, we are still a long way from fully understanding the neural circuitry of general anesthesia.

An explicit exploration of the role of specific neural circuits under general anesthesia requires advanced neural labeling and modulation technologies. In this article, three advanced techniques are reviewed: (1) in vivo calcium imaging, (2) chemogenetics, and (3) optogenetics. These three methods are currently applied to study the relationships between specific neural circuits and behavior in modern neuroscience research. In vivo calcium imaging records the activity of specific neurons and neural circuits in target brain regions [14], whereas the other two techniques artificially modulate the activity of specific neurons and neural circuits [15]. In the following sections, we will narrate examples of how these three techniques are employed in general-anesthesia research and highlight the merits and drawbacks of each method. This article aims to provide readers with the characteristics of these techniques and their applicability in study of anesthesiology.

## 2. In Vivo Calcium Imaging

Calcium ions are popular targets used to detect neuronal activities that link circuit dynamics to behaviors in modern neuroscience research [16,17]. Exploiting calcium ion properties, two main categories of calcium-ion indicators have been developed: chemical calcium dyes [18] and genetically encoded calcium indicators (GECIs) [19,20]. However, chemical calcium dyes, such as Fluo-4 and Oregon Green BAPTA-1 (OGB-1), are delivered through cell permeabilization, which can damage the cell integrity. Additionally, chemical calcium dyes are normally only capable of recording neuronal activity for several hours. These limitations constrain subsequent imaging conditions [21,22]. In contrast, GECIs could be easily expressed via virus-delivery methods, such as for adeno-associated-virus or lentivirus vectors, and cause the least cellular damage. Furthermore, GECIs could even be successfully expressed by transgenic methods without invasive procedures [23,24]. Thus, GECIs can be stably expressed in neurons and allow recording of the neural-firing patterns over a long period of time. Most importantly, GECIs are capable of selective yet unbiased labeling of neuronal types through their specific gene promoters, enabling research on the activities of each neuronal cell type. Moreover, GECIs can also be expressed at the nerve-projection terminals, so in vivo calcium imaging can directly monitor the activity of specific neural circuits [25]. Thus, in vivo calcium imaging has several advantages over traditional in vivo electrophysiological recording in studying neural endpoints and circuits under general anesthesia. Nowadays, GECIs, such as the GCaMP6s series, which possess a high time sensitivity and fluorescence signal-to-noise ratio [26], have become one of the most widely used calcium-ion detection tools [27,28].

**Figure 1:**
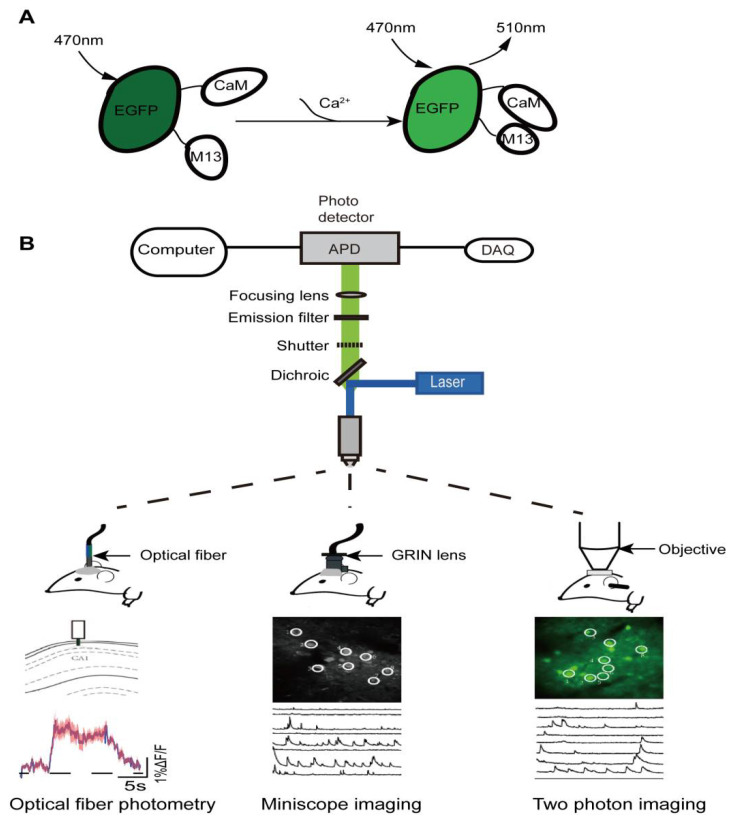
Schematic diagram of imaging principles of in vivo calcium imaging. (**A**) In basal condition: CaM and M13 are not bound to each other, and the fluorescence intensity of EGFP generally remains constant and low; in stimulated condition: the fluorescence intensity of EGFP increases significantly when calcium ions bind directly to EGFP. CaM, calmodulin; M13, calmodulin-binding peptide; EGFP, enhanced green fluorescent protein. (**B**) The schematic diagram of imaging device principles (**top**) and calcium-signal characterization for the three in vivo calcium imaging techniques (**bottom**).

GECIs bind to calcium ions and emit fluorescence signals that can be used to determine intracellular calcium concentration [20] (Figure 1A). These GECI-based signals can be detected by several methods, including optical-fiber photometry, miniscope imaging and two-photon imaging (Figure 1B). GECIs fluorescence is stimulated through an optical fiber in optical-fiber photometry. It has the advantages of not restricting animal movements and of monitoring deep-brain regions. Thus, optical-fiber photometry coupled with the GECI technology allows the activities of neurons and neural projections in any deep-brain area related to a specific behavior to be captured in freely moving animals [29]. Among the three in vivo calcium imaging technologies, optical-fiber photometry is currently the most widely used one in the study of neural-circuit mechanisms in general anesthesia. However, despite many advantages, optical-fiber photometry has its disadvantages. For example, compared with other in vivo calcium imaging techniques, its spatial resolution is relatively low, and it can only monitor changes in fluorescence intensity in neuron populations rather than at the single-neuron level. Therefore, it is difficult to detect relatively tiny excitability changes in cell groups using optical-fiber photometry. Further, the GECI-delivery methods, such as viral transfection or dyes, and the optical-fiber implantation, inevitably cause damage to the brain tissue, especially when targeting deep-brain regions.

Miniscope imaging has a high spatial resolution, and individual neuronal activity can be readily monitored [30]. The development of a head-mounted miniscope combined with Gradient Index (GRIN) lens-implantation technology also enables neural activities to be monitored in the deep-brain regions of freely moving animals [31]. However, viral transfection or lens implantation could also cause brain tissue damage. Additionally, this method is relatively more complex than fiber photometry in both surgical operation and imaging [32]. Two-photon microscopic-imaging technology with higher resolution is also applied, to identify the neural-population codes underlying complex brain functions [33]. Traditionally, the monitoring depth of this method is relatively shallow and generally limited to the study of neuronal activity in the cortex or hippocampus. In addition, it requires animal-head fixation, restricting the animal’s free movement [34,35]. These shortcomings may limit its wide application in the study of anesthesia neural circuitry to some extent. Fortunately, a newly developed miniature two-photon miniscope for large-scale calcium imaging in freely moving mice allows stable simultaneous recording of neuronal dynamics of densely active cortical regions in several behavioral tasks, without impediment to the animal’s behavior [36]. In summary, despite these limitations, with the continuous improvement of in vivo calcium imaging technology, it remains a valuable fundamental research technique with tremendous potential, especially in its application prospect in anesthesiology, such as the study of neural targets of analgesics and sedatives commonly used in the operating room.

In vivo calcium imaging technologies are widely applied to investigate whether specific neural endpoints or projections associated with the sleep–wake cycle participate in general anesthesia. The cortex is organized into six layers, and excitatory cells within each layer receive inputs from other excitatory cells in the same layer and from inhibitory cells. Simultaneously, the cerebral cortex receives a lot of subcortical projections, whose activity is critical for consciousness [37,38]. A recent two-photon calcium imaging study found that different anesthetics selectively synchronized activity in cortical pyramidal neurons within layer 5, which may contribute to the loss of consciousness induced by general anesthesia [39].

Monoaminergic neurons that drive arousal produce dopamine, serotonin, histamine, or norepinephrine, and they extensively innervate many brain regions. These monoaminergic cell groups share similar activity patterns, exhibiting high firing rates during wakefulness and slow firing rates during sleep [40]. The ventral tegmental area (VTA) mainly contains dopaminergic neurons and projects to abundant arousal-promoting brain areas [5]. Using real-time in vivo fiber photometry, researchers have shown that calcium signals of VTA dopaminergic neurons significantly decline after sevoflurane-induced loss of righting reflex (LORR) and robustly increase due to recovery of righting reflex (RORR) [41]. The nucleus accumbens (NAc) is situated in the ventral striatum and receives projections from dopaminergic neurons of VTA [42]. NAc neurons are similarly inhibited in the induction phase of propofol anesthesia and are markedly activated during recovery, and these effects are mediated by dopamine receptor 1 [41,43]. Moreover, the calcium signals of the ventral periaqueductal gray (vPAG) dopaminergic neurons also decrease during induction and increase during emergence, respectively, with isoflurane anesthesia [44]. Serotonergic (5-HTergic) neurons in the dorsal raphe nucleus (DRN), which project heavily to the midbrain and forebrain, are implicated in the modulation of the sleep–wake transition [45]. The calcium activity of DRN 5-HT neurons gradually declines after the initiation of isoflurane administration and begins to restore after the termination of anesthetics inhalation [46].

Together, these results suggest that similar to the role of promoting wakefulness during the sleep–wake cycle, the dopaminergic and serotonergic neurons in the brain may contribute to the consciousness transition that occurs during general anesthesia with various anesthetics. Two additional important awakening monoaminergic cell groups include the histaminergic neurons in the tuberopapillary nucleus and norepinephrine neurons in the LC. The actions of these neuron populations have not yet been reported using in vivo calcium imaging under general anesthesia. However, recently in a larval-fish model, a two-photon laser-based ablation study indicated that the LC neurons play a regulatory role in both the induction of and emergence from intravenous general anesthesia [47].

The basal forebrain (BF) is a heterogeneous region containing cholinergic, GABAergic, and glutamatergic neurons, and these BF neurons heavily innervate the cortex. In addition, it is worth noting that BF GABAergic neurons are functionally heterogeneous. Some BF GABAergic neurons are primarily active during the awake state, while others are more active during sleep. However, as a whole, BF plays a vital role in promoting quick cortical activity and arousal [48,49]. During both isoflurane and propofol anesthesia, calcium signals in BF cholinergic neurons decrease gradually during the induction period, begin to rise during the pre-awakening stage, and peak almost at the moment of RORR. Due to the reliable time accuracy and record stability advantages of optical-fiber photometry technology, researchers have observed that neuronal activity changes always precede behavioral changes. Therefore, BF cholinergic neuronal events may be the impetus for changes in the state of consciousness rather than the target of changes [50]. Given that the function of GABAergic BF neurons is clearly distinct in the sleep–wake cycle, it is odd that there have been no in vivo calcium imaging studies of these cell groups in general anesthesia. Future experiments are needed to target BF GABA neurons in both the induction to and emergence from general anesthesia.

Glutamatergic neurons in the parabrachial nucleus (PBN) are vital in contributing to both behavioral and cortical electroencephalogram arousal [51]. An optical-fiber photometry study in rats showed an obvious increase in the activity of PBN neurons during emergence from both isoflurane and propofol anesthesia but no significant change in the induction period [52]. On the other hand, the lateral habenula (LHb), another brain region clustered with glutamatergic neurons, plays an important role in promoting sleep but not arousal [53]. The average calcium activity of LHb is significantly increased during isoflurane anesthesia maintenance and begins to decline during RORR. Calcium signals of glutamatergic neurons in LHb show no change during the induction stage [54]. These interesting findings suggest that the glutamatergic neurons may play distinct roles in general anesthesia and the sleep–wake cycle. Compared with the dopaminergic and cholinergic systems, the glutamatergic system may only participate in the recovery phase, but not the induction phase of general anesthesia. Future research should focus on the responses of glutamatergic neurons and their projections in other brain nuclei during the induction phase of general anesthesia.

The lateral septum contains GABAergic neurons that project to multiple wakefulness-promoting subregions. The calcium activity of the dorsal–intermediate lateral septal GABAergic neuron changes in both the processes of induction and emergence, similar to the trend observed in the sleep–wake cycle [55]. Collectively, these in vivo calcium imaging findings suggest that multiple brain nuclei and neural circuits known to regulate the sleep–wake cycle play a similar role in general anesthesia (Table 1).

In vivo calcium imaging has recently been used to explore the neural responses and other effects of anesthetics and to study the neural-circuitry mechanisms during a loss of consciousness induced by general anesthesia. For example, Qiu et al. recently used fiber photometry to explore the role of the VTA in dexmedetomidine-induced sedation [56]. They demonstrated that selective activation of dopaminergic neurons in the VTA attenuates the depth of sedation in mice. Another study successfully employed optical-fiber photometry and miniscope technology to show that different general anesthetic drugs activate a shared population of central amygdala neurons to potently suppress pain reflexes [57]. Moreover, optical-fiber photometry and two-photon imaging have been successfully applied to study complications of brain dysfunction caused by different anesthetics [58,59]. Additionally, based on the principle of genetically encoded calcium ion indicators, several fluorescent proteins that can be used to characterize specific neurotransmitter concentrations have been developed, including the dopamine neurotransmitter probe [56], glutamatergic neurotransmitter probe [60], adenosine neurotransmitter probe [61] and orexin sensor probe [35]. These improvements provide a broader potential for the application of in vivo calcium imaging in many fields in the future.

## 3. Chemogenetics

In vivo calcium imaging can be used to determine the excitatory status of specific types of neurons or neural circuits in different brain areas under general anesthesia. However, it does not provide the ability to manipulate neural endpoints or circuits. In contrast, chemogenetics and optogenetics are capable of achieving this purpose.

Chemogenetics, as a technique similar to optogenetics, was developed earlier than optogenetic technology. It is based on genetic principles and utilizes small molecular tools to modulate the excitation or inhibition of target cells. This technology works by introducing engineered ligand-activated receptors into the neurons of targeted brain regions. Receptors are designed to be activated by specific exogenous ligands that are otherwise inert [15]. Since the design of a mutant β2-adrenergic receptor by Strader in 1991 [62], engineered receptors that respond specifically to synthetic small-molecule ligands rather than natural ligands have been refined and developed. For example, receptors activated solely by synthetic ligands (RASSLs) based on the κ-opioid receptor exhibit reduced binding affinity and signaling in response to dynorphin A(1–13) and 20 other opioid peptides, while maintaining a strong affinity and signaling in response to synthetic small-molecule agonists [63].

**Figure 2:**
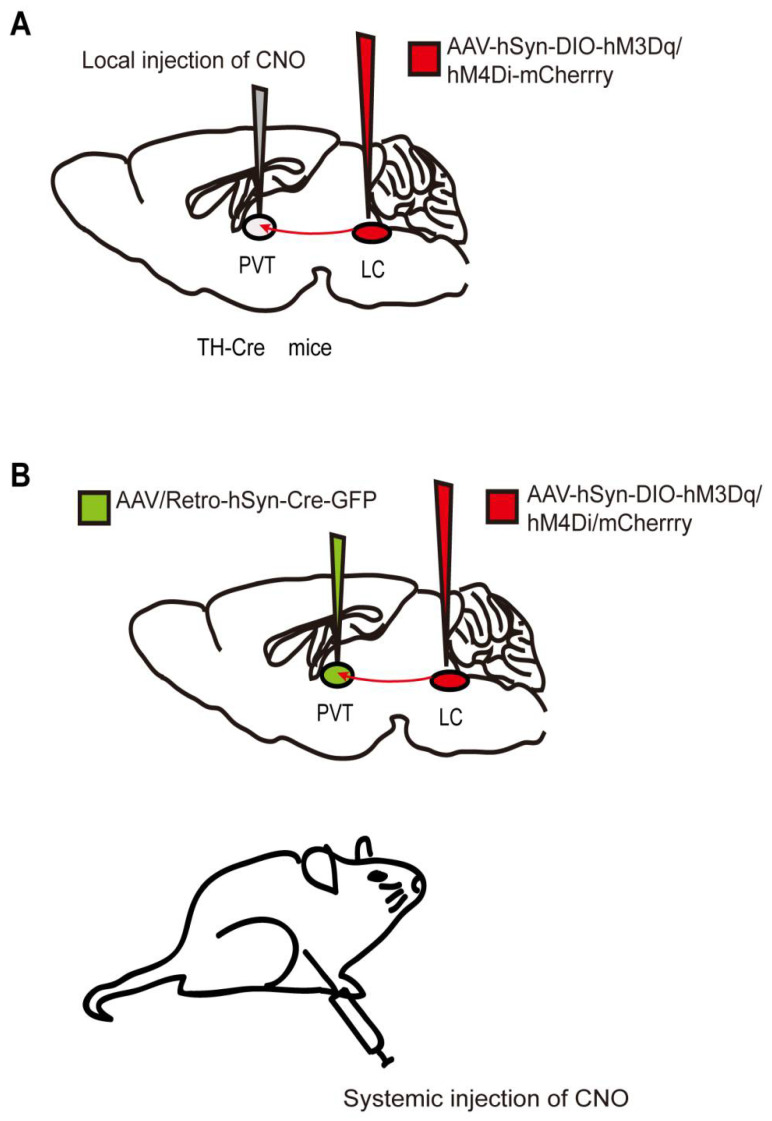
Two chemogenetic methods of specific neural-circuit manipulation. Taking the LC-PVT circuit as an example, (**A**) the AAV-hSyn-DIO-hM3Dq/hM4Di-mcherry was injected into the LC of TH-Cre transgenic mice, and CNO was injected locally in the PVT. (**B**) Wild-type mice were injected with the AAV-hSyn-DIO-hM3Dq/hM4Di-mcherry and AAV/Rtro-hSyn-Cre-GFP into the LC and PVT regions, respectively, followed by systematic injection of CNO.

The designer receptors exclusively activated by designer drugs (DREADDs) that are activated by specific exogenous drugs have been further developed since 2007 [64]. DREADDs, originating from human muscarinic receptors, are modified G-protein-coupled “designer” receptors. They are engineered with a low affinity for the native ligand but a high affinity for a synthetic inert “designer” ligand (e.g., clozapine-N-oxide, CNO) [64,65]. The most widely used DREADDs are hM3Dq and hM4Di, which produce excitatory and inhibitory effects, respectively, when CNO binds to the receptor. CNO binding to hM3Dq excites neurons by increasing intracellular calcium levels, whereas binding to hM4Di silences neuronal activity by reducing adenylate cyclase content [66]. In addition to the transgenic mouse lines expressing DREADDs [67], chemogenetic technology requires virus delivery to achieve specific and stable expression of hM3Dq or hM4Di in particular cells in one or more brain regions (Figure 2). Currently, DREADDs are widely applied to regulate specific neural activities and behaviors in many species, such as flies, mice, and even nonhuman primates [68,69]. DREADDs are the chemogenetic tools most widely used in the study of neural circuits under general anesthesia. Furthermore, a new κ-opioid-receptor-based inhibitory DREADD (KORD) has also been introduced. KORD is selectively activated by salvinorin B and is insensitive to endogenous opioid peptides [66]. In addition, KORD has been successfully applied to study the effects of rostromedial tegmental nucleus (RMTg) GABAergic neurons on nociception and opioid analgesia [70].

The most attractive features of chemogenetics are that it does not need intracranial implantation and that a single dose is sufficient to induce neural activation or inhibition in multiple brain regions for several hours. In addition, it has the advantages of relatively straightforward operation and simplicity compared to in vivo calcium imaging and optogenetics, as it does not require equipment such as that needed for calcium imaging or other laser-based methods [15]. However, its disadvantages do deserve attention. First, clozapine is a metabolite of CNO in the body. It is a sedative antipsychotic drug that interferes with the experimental results, mainly when CNO is administered at high doses [71]. Therefore, the current systemic quantity of CNO commonly used in laboratories is limited to 0.6–3 mg/kg. Second, when CNO is injected intraperitoneally, it activates or inhibits target neurons and all their projections. Therefore, study conclusions are ambiguous due to the fact that upstream regions of the brain may have opposite effects on different downstream regions. Lastly, the time accuracy of the chemogenetic method is insufficient, as the effects of CNO peak between 30 to 60 min after administration and last for about 9 h [68]. As long as these shortcomings are emphasized and used reasonably, this technology still has excellent potential in the field of anesthesiology.

Like NAc, the prelimbic cortex (PrL) is one of the vital projection regions of the VTA. VTA-NAc and VTA-PrL neural circuits are involved in both the induction and recovery periods during sevoflurane anesthesia, as evidenced by chemogenetic techniques [41,72]. By contrast, chemogenetic modulation of DRN 5-HT neurons shows that they are activated only during the recovery phase of general anesthesia [46]. These findings are inconsistent with the previous results obtained using optical-fiber photometry. This contradictory phenomenon indicates that artificially interfering with the activity of neural pathways may fail to fully mimic its normal physiological activity. LC is the primary source of norepinephrine in the brain and sends abundant outputs to many subregions of the forebrain, making it a vital arousal node [73]. Chemogenetic activation of LC in rats induces cortical arousal and a noticeable decrease in time to emergence from isoflurane, but the induction time remains unchanged [4,74]. Furthermore, the paraventricular thalamus (PVT) is a wakefulness-promoting region that receives numerous projections from the LC [75]. Researchers found that chemogenetic inhibition of LC-PVT projections significantly delayed emergence time from isoflurane anesthesia but had no impact on the induction phase [74]. Thus, although both dopaminergic and noradrenergic neurons belong to the class of monoaminergic neurons, there is a difference between these neurons in the process of consciousness transformation during general anesthesia. Dopaminergic neurons may be more integrally involved in the regulation of general anesthesia.

More interestingly, specific brain regions may have different regulatory effects based on the anesthetic used. For example, Luo et al. reported that when the PBN was activated by the chemogenetic method, it only accelerated the recovery times for propofol and isoflurane [52]. When using sevoflurane anesthesia, PBN glutamatergic neurons accelerated reanimation time and prolonged induction time [7]. Moreover, orexinergic neurons of the LHA, also known as hypocretin neurons, are vital for maintaining wakefulness and have numerous projections to many arousal-promoting brain areas [76,77]. A recent study showed that the actions of LHA orexinergic neurons and LHA-PVT are quite different regarding their actions during isoflurane vs. desflurane induction [78]. These unique phenomena suggest that different anesthetics may target specific neural pathways during the induction period, based on their specific pharmacological structures and physicochemical features. Furthermore, chemogenetic regulation of LHb glutamatergic neurons similarly shows that they contribute to the recovery time but not the induction time of general anesthesia [54]. In the cholinergic system, chemogenetic activation of BF cholinergic neurons affects both the induction time and the recovery time when using either isoflurane or propofol anesthesia, thereby attenuating the efficacy of general anesthesia [50]. Similar findings have been noted using both chemogenetic and in vivo calcium imaging studies, indicating that various wake-promoting brain nuclei or neural circuits are involved in the consciousness change caused by multiple anesthetics to varying degrees.

When exploring the role of neural pathways that promote sleep in general anesthesia, Jiang et al. identified multiple anesthetic-activated neurons in the hypothalamic preoptic area, an area traditionally viewed as a regulatory sleep center. Chemogenetic activation of these neurons reliably produces slow-wave sleep and facilitates general anesthesia, and chemogenetic inhibition shortens the general anesthesia time and disrupts natural sleep [9]. Recent studies have found that chemogenetic activation of GABAergic neurons in other brain regions, such as the VTA [79] and RMTg [80], promotes an anesthesia state as well, whereas chemogenetic activation of dorsal–intermediate lateral septum GABAergic neurons contributes to anesthesia emergence [55]. The fact that sleep-promoting GABAergic neurons in the aforementioned regions similarly contribute to anesthetic-state transitions further supports a common regulatory mechanism between the states of sleep and general anesthesia (Table 1). However, the role of other sleep-promoting brain nuclei and neural circuits in general anesthesia induced by different anesthetics needs further exploration.

## 4. Optogenetics

Optogenetic technology has gradually increased in popularity due to the shortcomings of chemogenetics, such as low timing accuracy. Many researchers combine both techniques to compensate for the disadvantages of each individual technique. Optogenetics regulates neurons by activating opsins expressed on target cells with a laser at the corresponding wavelength [15,81]. The light-sensitive opsins include excitatory and inhibitory types, usually channelrhodopsin (ChR2) and halorhodopsin (NpHR) or archaerhodopsin (Arch), respectively. Mutant receptors are employed to improve the reaction efficiency of opsins to light pulses or satisfy the requirements of the study. For example, activation of ChETAH, a ChR2 mutant, causes neurons to fire at up to 200 Hz, while wild-type ChR2 activation typically results in neurons firing at 20–40 Hz [82]. Therefore, ChETAH is specifically used to regulate neurons with higher firing frequencies. Similarly, red-shifted cruxhalorhodopsin has greater photocurrents than NpHR, making it possible for noninvasive photoinhibition due to the stronger penetrating potential of red light vs. blue light [83].

**Figure 3:**
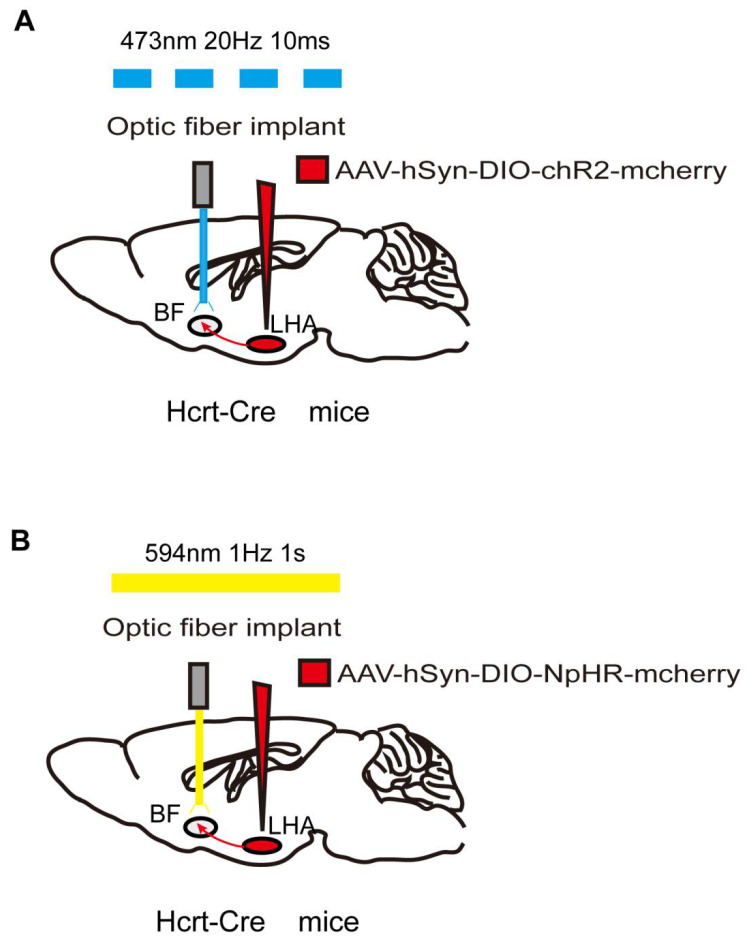
Optogenetic method of specific neural-circuit manipulation. Taking the LHA-BF neural circuit in Hcrt-Cre mice as an example, the opsin is typically introduced to the LHA neurons by injecting a virus containing ChR2 (**A**) or NpHR (**B**). After 3 weeks to allow for expression of the opsin, LHA-BF axon terminals can be targeted with corresponding light pulses from the optical fiber to excite (**A**) or inhibit (**B**) this pathway.

As with GECIs and DREADDs, in addition to the most commonly used viral strategies to transfect opsins into target neurons, transgenic mice expressing ChR2 have also been developed [84]. Traditionally, optogenetic technology requires optical-fiber implantation in target sites. Once implanted, optical fibers activate ChR2 or NpHR by adjusting the pulsed light using various specific parameters (Figure 3). The parameter settings, including the duration, frequency, and intensity of the light pulse, are based on the physiological firing pattern and rate of the target neurons. Artificial real-time control of receptor activation–deactivation and the duration of the light pulse allow optogenetics to activate or inhibit neurons with millisecond timescale precision [85]. Thus, optogenetics provides the powerful ability to modulate specific neurons or neural circuits with a precise temporal resolution.

However, there are several limitations in the use of photogenetic technology and the interpretation of its results. First, brain tissue may be damaged during fiber implantation. Fortunately, this problem has long been recognized and has been gradually solved by scientists. For example, Zhang et al. developed a wireless photogenetic technique mediated by upconversion nanoparticles (UCNPs). This technology converts near-infrared light to high-energy blue light via UCNPs, activating common photosensitive proteins such as ChR2 to stimulate deep-brain regions [86]. Recently, Gong et al. designed a new step-function opsin with ultra-high light sensitivity (SOUL), allowing for transcranial stimulation of neurons in the deep-brain regions of mice. SOUL is capable of regulating neuronal spiking in the macaque cortex via optical stimulation from outside the dura [87]. This non-invasive advantage, which does not require fiber implantation, makes these new optogenetic tools much less damaging and, thus, promising for future research.

A second limitation of photogenetic technology is the possibility of false-negative results. Light energy may be partially lost when passing through optical fibers, brain tissues, etc. Additionally, viral transfection may not be effective enough, resulting in a subset of target cells lacking opsins. These factors may confound the interpretation of negative behavioral results. Moreover, prolonged light stimulation may increase the temperature of the target tissue and cause an unexpected neurophysiologic reaction. Specifically, photoinhibition requires continuous receptor stimulation to suppress the spontaneous excitation of neurons when silencing neurons [88]. Furthermore, many types of neurons typically undergo an elastic increase in firing at the end of a long period of photoinhibition, which potentially leads to confounding results [89].

Finally, immunolabeling (e.g., c-fos protein expression) or whole-cell patch-clamp techniques are often used to verify illumination-induced excitatory or inhibitory responses at the cellular level. This verification step is also necessary for chemogenetics. Additionally, histological verification is also essential for the three techniques described in this review. For example, the position of the optical fiber in optogenetics and fiber photometry and the expression of the corresponding protein in the targeted brain regions are determined by fluorescence imaging and immunolabeling.

Consistent with chemogenetic results, optogenetic activation of orexinergic terminals in the PVT induces similar changes in induction to and emergence from desflurane and isoflurane anesthesia [78]. Dong et al. further found that selective light stimulation of LHA orexinergic neurons and their projections to BF, LC, and VTA resulted in a shorter emergence time from isoflurane anesthesia in rats. In contrast, optogenetic inhibition of orexinergic terminals in the VTA delayed the time to wakefulness. These studies failed to detect a significant difference in induction time [90,91]. Whether the downstream nuclei of LHA orexinergic neurons play a role in the induction phase with other anesthetics remains to be further elucidated. As observed in chemogenetic studies, optical stimulation of VTA dopaminergic neurons [5] and VTA-NAc and VTA-PrL pathways [41] contributes to the transition of consciousness during both the induction and emergence phases of isoflurane or sevoflurane general anesthesia. Optical stimulation of LC TH axons in the PVT does not alter the induction time but does elicit emergence from 1.2% isoflurane [74]. Furthermore, it has been demonstrated that the brain’s dopaminergic system may play a more important role than its noradrenergic system in determining the effects of general anesthesia.

Using optogenetic tools, Wang et al. aimed to elucidate further the role of glutamatergic neurons of the PBN in emergence from sevoflurane anesthesia. They unexpectedly found that photostimulation of PBN neurons only caused cortical arousal and did not lead to significant changes in behaviors [7]. The authors concluded that this phenomenon was due to the limitations of optogenetic technology, such as the absorption, scattering, and distance-related attenuation of light passing through the brain tissue, and the decay of the light subsequently transmitted to the target area [92]. Optogenetics has also been employed to investigate the specific role of glutamatergic neurons in the LHA. For example, light stimulation of LHA glutamatergic neurons reduces the depth of isoflurane-inhalation anesthesia, and light activation of LHA glutamatergic projections to the LHb accelerates the recovery time from isoflurane anesthesia [93].

Optogenetics has also been used to identify sleep-promoting nuclei and neural circuits activated or suppressed during general anesthesia. Jiang et al. reported that optogenetic activation of anesthetics-activated neurons in the hypothalamus preoptic area accelerates sleep and enhances the effects of general anesthesia. In contrast, optogenetic inhibition of these neurons reduced the duration of general anesthesia [9]. Again, these results demonstrate that the hypothalamic preoptic area plays a crucial role in maintaining general anesthesia. After optogenetic activation of the LHb containing glutamatergic neurons [54] and VTA-LHA GABAergic projections, behavioral studies have shown that LORR significantly declines and RORR statistically increases. Optical inhibition of VTA-LHA GABAergic projections induces the opposite effects in both the EEG and behavioral outcomes [79]. The results of these optogenetic studies are similar to previous findings from in vivo imaging and chemogenetic studies, which suggest that despite anesthesia and sleep sharing some overlapping neural pathway mechanism, there are many differences that are anesthetic-specific (Table 1).

## 5. Discussion

**Figure 4:**
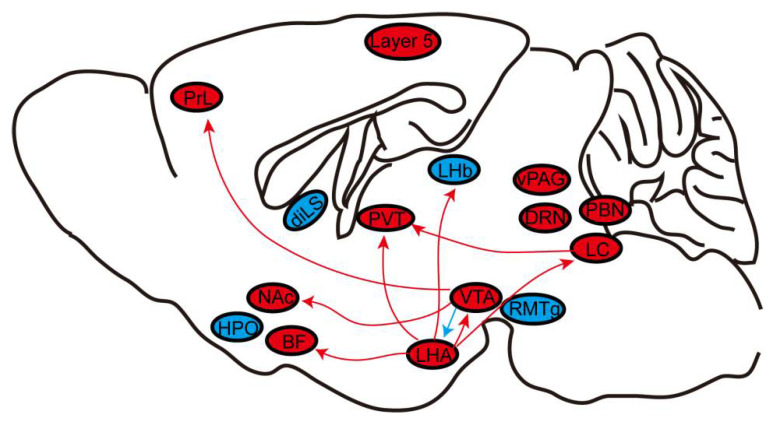
The role of neural nuclei and circuits under general anesthesia. General anesthesia generally involves silencing wake-active nuclei or circuits (red) and stimulating sleep-active nuclei or circuits (blue). BF, basal forebrain; diLS, dorsal–intermediate lateral septum; DRN, dorsal raphe nucleus; HPO, hypothalamus preoptic area; LC, locus coeruleus; LHA, lateral hypothalamus area; LHb, lateral Habenula. NAc, nucleus accumbens; PBN, parabrachial nucleus; PrL, prelimbic cortex; PVT, paraventricular thalamus; RMTg, rostromedial tegmental nucleus; vPAG, ventral periaqueductal gray; VTA, ventral tegmental area.

Since the application of general anesthesia to clinical practice, its mechanism of action has been investigated for many decades. In recent years, thanks to the rapid development of experimental technologies, research has increasingly focused on the role of neuroanatomic sites or pathways related to sleep in promoting or inhibiting the effects of general anesthesia [94]. Advances in neuroscience fields, such as in vivo calcium imaging, chemogenetics, and optogenetics, coupled with the advantages of unambiguously labeling neuronal types through genetic strategies, have provided support for detailed studies of particular neurons or neural circuits under general anesthesia. To date, using the three advanced techniques reviewed here, scientists have discovered that many wakefulness-promoting and several sleep-promoting nuclei play essential regulatory roles in general anesthesia (Figure 4). Thus, current studies mainly focus on the role of the wakefulness-promoting neural nuclei or pathways in general anesthesia. By contrast, there are relatively few studies on the neural endpoints or pathways that promote sleep, such as VLPO, the median preoptic nucleus, and their projections [95], as well as GABAergic neurons in the BF [96] or the parafacial zone [97], all of which have been identified as sleep-active neurons. These sleep-promoting brain regions and other wake-promoting neural endpoints and circuits that have not yet been reported should be the focus of future research.

Furthermore, on one hand, the actions of different neural nuclei and circuits during the induction phase of specific anesthetics vary. On the other hand, specific brain regions also respond differently to different anesthetics. In addition to possessing different pharmacological properties, it is also possible that different anesthetics may not share the same neural circuits. This phenomenon may explain why anesthetic effectiveness varies among the different analgesics, such as the shorter recovery time observed for desflurane and sevoflurane compared to isoflurane. Therefore, despite some significant progress, we are still far away from fully understanding the mechanism of general anesthesia. Future research may focus on the specific effects of various analgesics on the same neural nuclei or circuits.

Finally, three powerful experimental tools have been extensively applied to the study of anesthesia and other research topics, however, each technique has its limitations. Only by fully understanding their strengths and weaknesses can scientists apply them more effectively. Fortunately, some limitations have already been recognized and addressed. With the rapid development of science and technology, these three methods will likely to be improved in the future. In addition, hopefully, new experimental technologies will appear as well that will help researchers gradually uncover the mechanism of general anesthesia and solve clinical-anesthesia-related problems.

## Figures and Tables

**Table 1:** Main findings with the three technologies in the neural nuclei and circuits of general anesthesia.

Neuron Type of Brain Region and Its Projections	Technology	Anesthetic Method	Experimental Animals (Numbers)	The Role of Induction to or Emergence from General Anesthesia
Layer 5 cortical pyramidal neurons	In vivo two-photon calcium imaging	Isoflurane, Fentanyl-Medetomidine-Midazolam, and Ketamine-Xylazine	Rbp4-cre mice (22)	Both
VTA dopaminergic neurons and VTA-NAc and VTA-PrL dopaminergic projection	Optical-fiber photometry, optogenetics and chemogenetics	Sevoflurane	DAT-cre mice (64); Rats (67)	Both
NAc neurons and NAc GABAergic neurons	Optical-fiber photometry	Sevoflurane and propofol	Mice (18); Rats (12)	Both
vPAG dopaminergic neurons	Optical-fiber photometry	Isoflurane	Rats (12)	Both
DRN 5-HT neurons	Optical-fiber photometry	Isoflurane	Sert-cre mice (6)	Both
	Chemogenetics		Sert-cre mice (24)	Emergence
LC TH neurons and LC-PVT	Chemogeneticsand optogenetics	Isoflurane	Rats (32); TH-cre mice (54)	Emergence
BF cholinergic neurons	Optical-fiber photometry and chemogenetics	Isoflurane and propofol	ChAT-cre mice (40)	Both
PBN glutamatergic neurons	Optical-fiber photometry, Chemogeneticsoptogenetics	Isoflurane and propofol	Rats (42)	Emergence
		Sevoflurane	Vglut2-cre mice (32)	Both
LHb glutamatergic neurons	Optical-fiber photometry, chemogenetics, optogenetics	Isoflurane	Vglut2-cre mice (68)	Emergence
LHA glutamatergic neurons and LHA-LHb glutamatergic projection	Optogenetics andchemogenetics,	Isoflurane	Vglut2-cre mice (48)	Emergence
LHA orexinergic neurons and LHA-PVT orexinergic projection	Chemogenetics and optogenetics	Isoflurane	Hcrt-cre mice (83)	Emergence
		Desflurane	Hcrt-cre mice (83)	Both
LHA orexinergic neurons, LHA-BF, LHA-LC, and LHA-VTA orexinergic projections	Optogenetics	Isoflurane	Hcrt-cre mice (69)	Emergence
Dorsal–intermediate lateral septum GABAergic neurons, and dorsal–intermediate lateral septum-VTA GABAergic projection	Optical-fiber photometry, chemogenetics and optogenetics	Isoflurane	Vgat-cre mice (56)	Both
Hypothalamus preoptic area’s GABAergic neurons	Chemogeneticsand optogenetics	Isoflurane, propofol, and ketamine	Mice (39)	Emergence
VTA GABAergic neurons, and VTA–LHA GABAergic projections	Chemogenetics	Isoflurane	Vgat-cre mice (30)	Both
RMTg GABAergic neurons	Chemogenetics	Sevoflurane	Vgat-cre mice (18)	Both
